# Supplementary material for: Seroprevalence of anti-Toxoplasma gondii antibodies in wild boars (Sus scrofa), hunting dogs, and hunters of Brazil
Source: PLoS One. 2019 Oct 11;14(10):e0223474. doi: 10.1371/journal.pone.0223474 (PMC6788692; doi:10.1371/journal.pone.0223474)
Supplement: Attachment — Submitted filename: review_D_19_16414.docx [file pone.0223474.s002.docx]

**Concomitantly serosurvey of anti-*Toxoplasma gondii* antibodies in wild boars (*Sus scrofa*), hunting dogs and hunters of Brazil**

The title could be improved:

**Seroprevalence of anti-*Toxoplasma gondii* antibodies in wild boars (*Sus scrofa*), hunting dogs and hunters from the southern Brazil.of Brazil**

**Line 85**, change the sentence *Toxoplasma gondii* is considered the most widespread coccidian worldwide.

Please use the most up-to-date bibliography of the same author. For example.

Dubey, J.P., 2010. Toxoplasmosis of Animals and Humans, Second Edition. CRC Press, Boca Raton, Florida, 313 pp. Hill, D.E., Dubey, J.P., 2015. Toxoplasma gondii. Biology of Foodborne Parasites. CRC Press. 209-222 pp.

**Line 86**, “warm-blood” it is not a scientific term.

**Line 86**, change the sentence Felids are the definitive hosts and produce the environmentally resistant oocyst stage, while many mammals and avian species are intermediate hosts. Asexual stages of *T. gondii* found in intermediate host tissues may become an infection source, particularly by ingestion of raw or undercooked meat.

Please use the most up-to-date bibliography of the same author. For example.

Hill, D.E., Dubey, J.P., 2015. Toxoplasma gondii. Biology of Foodborne Parasites. CRC Press. 209-222 pp.

**Line 91**, what are you referring to with their hybrids? Wild pigs?

**Line 90-93**, not clear what the authors are trying to state. Despite is a contrast connector.

In Brazil, as in other countries of South America, wild boar (*Sus scrofa*) is an exotic invasive species. Its presence produces strong negative impact on health, livestock production and native wildlife. As a strategy to control its population, the hunting of wild boar is allowed

**Line 94**, why you said “As hunting dogs have been frequently used by Brazilian hunters in wild boar hunting, dogs may be also directly exposed to *T. gondii* infection [4]” if in the abstract you said “Surprisingly, no toxoplasmosis serosurvey has been eported to date in wild boar hunters and their hunting dogs”? particularly when consuming raw wild boar meat.

The paragraph is confusing. Please change the paragraph

In Brazil, as in other countries of South America, wild boar (*Sus scrofa*) is an exotic invasive species. Its presence produces strong negative impact on health, livestock production and native wildlife. Wild boar hunting is allowed as a strategy to control it population. Wild boars are hunted by hunting teams with several dogs. So dogs are also directly exposed to *T. gondii* infection too.

**Line 96**, please change serosurveys for seroprevalence or *T. gondii* infection. Change in all the paper.

**Line 97-121**, the paragraph is very long. Each author of each of these prevalence found must be cited. But, as the study area is a South American country, they could choose to mention prevalence of this continent with more emphasis and then some (more current) of other countries of Europe and Asia. Consider the investigations that have used the same technique.

Rewrite in one paragraph.

**Line 131**, Meat from infected animals is considered the most important source of *T. gondii* human infections. Hill, D.E., Dubey, J.P., 2015. Toxoplasma gondii. Biology of Foodborne Parasites. CRC Press. 209-222 pp.

**Line 133 to 137**, should go after the line in the same paragraph.

**Line 133**, change serosurveys for seroprevalence and fauna for wildlife please in all the manuscripts.

**Line 136-137**, what domestic animals are referred to in this sentence? because dogs have already been mentioned. Write two separate sentences for the results in pets and humans please.

**Line 138-139**, it is very similar to Line 131. Who says it?

Lack of bibliography

**Line 141**, prevalence of antibodies to *T. gondii.* The authors did not detect the infection, they detected the presence of antibodies whose presence

does not always coincide with the presence of the pathogen.

**Line 141**, the use of Accordingly is not necessary, please deleted.

The present cross-sectional study was conducted in the Campos Gerais National Park and periruban areas of Curitiba, Castro, Palmeira, Ponta Grossa, Porto Amazonas, and Teixeira Soares.

Then you can include a description of area: vegetation, climate…

In the Figure1 please delete the word “only” from references. Dog samples, humans samples it is ok. But include biomas in the figure title.

The Municipalities: blue, red, violet and light blue areas what kind of biome have? That information is not clear in the Figure.

**Line 162**, serum samples of wild boars, hunting dogs, and hunters were collected.

**Line 163**, ~~during 22 on-field expeditions carried out~~ ~~from~~ between October 2016 and May 2018.

**Line 166**, without anti-coagulant and kept at ~~room temperature~~ 25°C.

**Line 166-168**, until visible retraction of the clot, centrifuged five minutes at 1,500 rpm. The serum was separated and kept at -20 ° C until analyzes.

**Line 181-188**, authors should provide more detail on the performance metrics of the IFAT (sensitivity, specificity).

The subtitle is “Serum Sample Collection”. Was any other type of sample collected? Change the subtitle to Sample collection.

Serological diagnosis change to Antibody detection or Serological test.

Data analysis change to Epidemiological data collection and separate in another subtitle Statistical analysis.

**Line 191-194**, please write separately and what information was collected from the wild boars, dogs and people. Use separate sentences.

**Line 199**, *T. gondii*

**Line 158** (Fig 1) and **Line 208** (Figure 1) ??

**Line 206**, correct please bioma for biome in all the manuscripts and the figure.

**Line 225**,

Antibodies to *T. gondii* were detected in 15/71 (21.1% - CI 95% 12.7-32.6%) wild boars, 49/157 (31.2% -226 CI 95% 24.8-38.3%) hunting dogs and 15/49 (32.7% - CI 95% 21.7-47.1%) 227 hunters.

**Line 227**, *T. gondii*

**Line 234**, “Seropositivity for T. gondii was statistically higher in 12/14 (85.7%) 235 captured wild boars when compared to 5/57 (7.0%) free-range wild boars”. What the difference between captured and free-range wild boars?? explain it in Sample collection please.

**Line 243**, change “hunting dogs were not statistically significant between males and females (p=0.135)”…

**Line 244**, please deleted gender and use sex.

**Line 258**, please start the paragraph “The statistical analysis showed …” for a correct wording in English.

**Line 268**, meat (p=0.024) and raw fish (p=0.026) were statistically 268 significant ~~and protective.~~

**Line 272**,

“To the authors knowledge, the present study has been the first concomitant report of wild boar, hunting dog, and hunter”.

The authors must make an adequate review for the work, it cannot be simply an appreciation. You can write:

This is the first study that reveals the presence of antibody to *T. gondii* in wild boars, hunting dogs and hunters and carries out an epidemiological risk analysis.

“performed herein in southern and central-western regions of Brazil”.

Is the first study in Brazil? in that region of Brazil?

**Line 276**, the seroprevalence obtained to *T. gondii* antibodies in the present study is higher than….

**Line 277-282**, how is the comparison with studies in the South America? Could wild boar with antibodies to *T. gondii* expand to other countries that have not positive to *T. gondii* wild boar?

**Line 328**, please deleted herein in all de manuscript.

The authors should discuss why they used MAT for wild boars and IFAT for hunting dogs and hunters.

In the discussion they should highlight in cases of significant statistical differences and discuss what they should be.

Why the title of the hunting dogs is higher? to what could it be?

The authors could discuss about wild boars feeding habits.

**Line 345-351**, I do not agree that in the last paragraph there is reference to another pathogen that is not the one that motivates this investigation. Please deleted or rewrite.

**Line 356-357**, this sentence is inconsistent. You cannot make that claim with 71 wild boar and 157 hunting dog serum samples.

**Line 358-362**, this sentence is inconsistent. You cannot make that claim. Wild boars are able to travel kilometers in just one night.

Final comments

The research and the results obtained are relevant because published works of this subject in South America are reduced, while wild boar population continues to grow and expand by the continent.

- The manuscript needs editing for English.

- There are also some statements that are not clear as written.

- The authors should deepen the discussion.

The study is important to be published with the pertinent corrections.
