# Supplementary material for: Seroprevalence of anti-Toxoplasma gondii antibodies in wild boars (Sus scrofa), hunting dogs, and hunters of Brazil
Source: PLoS One. 2019 Oct 11;14(10):e0223474. doi: 10.1371/journal.pone.0223474 (PMC6788692; doi:10.1371/journal.pone.0223474)
Supplement: Attachment — Submitted filename: Response to reviewers 21thAugust2019.docx [file pone.0223474.s003.docx]

August 17^th^, 2019

**Response to reviewers**

To: Paulo Lee Ho, PhD / Academic Editor - PLOS ONE

Re: author’s PLOS ONE manuscript resubmission (PONE-D-19-16414)

Dear Dr. Ho,

Please find attached our reviewed manuscript entitled "*Concomitantly serosurvey of anti-Toxoplasma gondii antibodies in wild boars (Sus scrofa), hunting dogs and hunters of Brazil*" (PONE-D-19-16414). All the corrections and suggestions indicated by the reviewers were fully accepted, addressed and rewritten.

A letter has been written to pinpoint the reviewers’ requests, with a detailed list of corrections and a description of changes made within the manuscript. To better identify the corrections of each reviewer, changes were highlighted in different colors in the revised manuscript.

Thank you for the opportunity and please don’t hesitate to contact us with any further question.

Sincerely,

Alexander Welker Biondo, DMV, MSc, PhD.

Professor, Department of Veterinary Medicine, UFPR, Brazil

Visiting Professor, Purdue University, IN, USA

E-mail: [abiondo@ufpr.br](mailto:abiondo@ufpr.br) / Phone: +55 (41) 3350-5812

**Manuscript PONE-D-19-16414**

**Title:** "Concomitantly serosurvey of anti-*Toxoplasma gondii* antibodies in wild boars (*Sus scrofa*), hunting dogs and hunters of Brazil."

**Dear Reviewer #1,**

Firstly, we appreciate all your suggestions and comments and our manuscript. Certainly, your suggestions were very important to improve the manuscript.

**1. Reviewer #1 (green intext):**

Comments to the Author

-1. Is the manuscript technically sound, and do the data support the conclusions?

Reviewer #1: Yes

-2. Has the statistical analysis been performed appropriately and rigorously?

Reviewer #1: Yes

-3. Have the authors made all data underlying the findings in their manuscript fully available?

Reviewer #1: Yes

-4. Is the manuscript presented in an intelligible fashion and written in standard English?

Reviewer #1: Yes

Review Comments to the Author

1.1. Concomitantly serosurvey of anti-Toxoplasma gondii antibodies in wild boars (*Sus scrofa*), hunting dogs and hunters of Brazil. The title could be improved:

Seroprevalence of anti-*Toxoplasma gondii* antibodies in wild boars (*Sus scrofa*), hunting dogs and hunters from the southern Brazil.of Brazil

**Changed:** Reviewer is right. The title has been changed.

**Now you read:** “Seroprevalence of anti-*Toxoplasma gondii* antibodies in wild boars (*Sus scrofa*), hunting dogs and hunters of Brazil” (Page 1, Lines 1-2).

1.2. Line 85, change the sentence *Toxoplasma gondii* is considered the most widespread coccidian worldwide. Please use the most up-to-date bibliography of the same author. For example.

Dubey, J.P., 2010. Toxoplasmosis of Animals and Humans, Second Edition. CRC Press, Boca Raton, Florida, 313 pp.

Hill, D.E., Dubey, J.P., 2015. Toxoplasma gondii. Biology of Foodborne Parasites. CRC Press. 209-222 pp.

**Changed:** The sentence has been changed and the suggested bibliography added, improving an updated discussion.

**Now you read:** “*Toxoplasma gondii* is a coccidian parasite relying on cats and other *Felidae* as definitive hosts, which may shed fecal oocysts that can infect a variety of homeothermic animals as intermediate hosts [1, 2]. Since infected intermediate hosts may harbor viable tissue cysts for years, human beings may be infected by ingestion of contaminated raw or undercooked meat [2, 3].” (Page 4, Lines 85-89).

“Native ocelots and mountain lions were the only felids present within the state park limits; these species have no confirmed capacity to be definitive hosts or shed oocysts, suggesting a low in-park *Toxoplasma* circulation due to a single (5.0%) seropositive wild boar.” (Page 3, 58-61).

“On the other hand, no in-park pets have been allowed in the state park area, and only a dozen ocelots (*Felis pardalis*) and a couple of mountain lions (*Puma concolor*) were observed within the state park limits; these felid species have not yet been confirmed as *Toxoplasma* definitive hosts or capable of shedding oocysts [1]. Thus, the only 1/20 (5.0%) seropositive wild boar likely reflects the low *Toxoplasma* in-park circulation.” (Page 17, 318-323).

1.3. Line 86, “warm-blood” it is not a scientific term.

**Changed:** The “warm-blood” has been changed for “homeothermic”.

**Now you read:** “*Toxoplasma gondii* is a coccidian parasite relying on cats and other *Felidae* as definitive hosts, which may shed fecal oocysts that can infect a variety of homeothermic animals as intermediate hosts [1, 2].” (Page 4, Lines 85-87).

1.4. Line 86, change the sentence Felids are the definitive hosts and produce the environmentally resistant oocyst stage, while many mammals and avian species are intermediate hosts. Asexual stages of *T. gondii* found in intermediate host tissues may become an infection source, particularly by ingestion of raw or undercooked meat. Please use the most up-to-date bibliography of the same author. For example.

Hill, D.E., Dubey, J.P., 2015. Toxoplasma gondii. Biology of Foodborne Parasites. CRC Press. 209-222 pp.

**Changed:** The paragraph has been changed.

**Now you read:** “*Toxoplasma gondii* is a coccidian parasite relying on cats and other *Felidae* as definitive hosts, which may shed fecal oocysts that can infect a variety of homeothermic animals as intermediate hosts [1, 2]. Since infected intermediate hosts may harbor viable tissue cysts for years, human beings may be infected by ingestion of contaminated raw or undercooked meat [2, 3]. “(Page 4, Lines 85-89).

1.5. Line 91, what are you referring to with their hybrids? Wild pigs?

**Changed:** Wild boars hybrids have been described as the result of reproductive crossing with domestic pigs (*Sus scrofa domesticus*). The above information on hybrids has been deleted, and reviewer suggestion has been added.

**Now you read:** “In Brazil, as in other South American countries, wild boar (*Sus scrofa*) is an exotic invasive species [4]. Its presence produces a substantial negative impact on health, livestock production, and native wildlife [5]. Wild boar hunting has been allowed as a strategy to control their population [5]. Hunters are organized in teams commonly accompanied by several hunting dogs [6]. Thus, dogs are also directly exposed to *T. gondii* infection.” (Page 5, Lines 90-95).

1.6. Line 90-93, not clear what the authors are trying to state. Despite is a contrast connector.

In Brazil, as in other countries of South America, wild boar (*Sus scrofa*) is an exotic invasive species. Its presence produces strong negative impact on health, livestock production and native wildlife. As a strategy to control its population, the hunting of wild boar is allowed.

**Changed:** Reviewer is right. Sentence has been changed to better explain it.

**Now you read:** “In Brazil, as in other South American countries, wild boar (*Sus scrofa*) is an exotic invasive species [4]. Its presence produces a substantial negative impact on health, livestock production, and native wildlife [5]. Wild boar hunting has been allowed as a strategy to control their population [5]. Hunters are organized in teams commonly accompanied by several hunting dogs [6]. Thus, dogs are also directly exposed to *T. gondii* infection.” (Page 5, Lines 90-95).

1.7. Line 94, why you said “As hunting dogs have been frequently used by Brazilian hunters in wild boar hunting, dogs may be also directly exposed to *T. gondii* infection [4]” if in the abstract you said “Surprisingly, no toxoplasmosis serosurvey has been reported to date in wild boar hunters and their hunting dogs”? particularly when consuming raw wild boar meat.

The paragraph is confusing. Please change the paragraph

In Brazil, as in other countries of South America, wild boar (Sus scrofa) is an exotic invasive species. Its presence produces strong negative impact on health, livestock production and native wildlife. Wild boar hunting is allowed as a strategy to control it population. Wild boars are hunted by hunting teams with several dogs. So dogs are also directly exposed to *T. gondii* infection too.

**Changed:** The paragraph has been changed to better explain it, following the reviewer suggestion.

**Now you read:** **“**In Brazil, as in other South American countries, wild boar (*Sus scrofa*) is an exotic invasive species [4]. Its presence produces a substantial negative impact on health, livestock production, and native wildlife [5]. Wild boar hunting has been allowed as a strategy to control their population [5]. Hunters are organized in teams commonly accompanied by several hunting dogs [6]. Thus, dogs are also directly exposed to *T. gondii* infection.” (Page 5, Lines 90-95).

“Surprisingly, the seroprevalence of toxoplasmosis has not been reported to date in wild boar hunters and their hunting dogs.” (Page 2, Lines 37-38).

1.8. Line 96, please change serosurveys for seroprevalence or *T. gondii* infection. Change in all the paper.

**Changed:** Serosurvey has been changed for seroprevalence throughout the manuscript.

**Now you read:** “Seroprevalence of *T. gondii* has been extensively studied in free-range wild boars throughout the world [7].” (Page 5, Lines 96-97).

“Interestingly, another seroprevalence study in the same nearby city [20] reported higher exposure in non-domiciled dogs, with 175/364 (48.1%) positivity, which reemphasizes the importance of microenvironment and pet ownership on dog seroprevalence.” (Page 5, 114-118).

“Although seropositivity to *T. gondii* has also varied in Brazil, all previous studies have shown lower prevalence, either in farmed or free-range wild boars [38, 39].” (Page 16, 299-300).

1.9. Line 97-121, the paragraph is very long. Each author of each of these prevalence found must be cited. But, as the study area is a South American country, they could choose to mention prevalence of this continent with more emphasis and then some (more current) of other countries of Europe and Asia. Consider the investigations that have used the same technique. Rewrite in one paragraph.

**Changed:** Reviewer is right. The paragraph has been shortened.

**Now you read:** “Seroprevalence of *T. gondii* has been extensively studied in free-range wild boars throughout the world [7]. In South America, Argentina has recently reported the presence of antibodies to *T. gondii* in 18/144 (12.5%) free-range wild boars [8], whereas, in Brazil, the positivity reported was 14/306 (4.5%) in young farmed animals and 5/34 (14.28%) in free-range wild boars [9]. Another study in Brazil reported the seropositivity of 0/7 (0.0%), 16/101 (15.8%), and 3/14 (21.4%) in free-range wild boars from different regions [10], corroborating with the worldwide in-country variation on *T. gondii* exposure. In European wild boars, the seroprevalence of *T. gondii* ranges from 10/150 (6.7%) in Switzerland to 8/8 (100%) in Portugal [11, 12]. In Asia, the reported ranges are from 1/90 (1.1%) in Japan to 152/426 (35.6%) in South Korea [13,14]. Lastly, in North America, seropositivity has been reported from 34/376 (9.0%) to 181/227 (49.0%) in free-range wild boars from the United States [15, 16].” (Page 5, Lines 96-108).

1.10. Line 131, meat from infected animals is considered the most important source of *T. gondii* human infections. Hill, D.E., Dubey, J.P., 2015. *Toxoplasma gondii*. Biology of Foodborne Parasites. CRC Press. 209-222 pp.

**Changed:** Reviewer is right. The sentence has been changed and the bibliography added.

**Now you read:** “Meat from infected animals is considered the most important source of *T. gondii* human infections [2], including the consumption of exotic or native free-range species [20,21].” (Page 5-6, Lines 119-121).

1.11. Line 133 to 137: Should go after the line in the same paragraph.

**Changed:** The sentence has been continued after the same paragraph.

**Now you read:** “Meat from infected animals is considered the most important source of *T. gondii* human infections [2], including the consumption of exotic or native free-range species [20,21]. Among wildlife species used for hunting, seroprevalence on native Brazilian species revealed *T. gondii* seropositivity in 4/21 (19.0%) of free-range and 1/10 (10.0%) captive capybaras, along with 7/22 (31.8%) captive collared peccaries [22].” (Page 5-6, 119-124).

1.12. Line 133, change serosurveys for seroprevalence and fauna for wildlife please in all the manuscripts.

**Changed:** Seroprevalence and wildlife have been inserted throughout the manuscript.

**Now you read:** “Among wildlife species used for hunting, seroprevalence on native Brazilian species revealed *T. gondii* seropositivity in 4/21 (19.0%) of free-range and 1/10 (10.0%) captive capybaras, along with 7/22 (31.8%) captive collared peccaries [22].” (Page 6, Lines 121-124).

“Despite the potential as sentinels of environmental toxoplasmosis, seroprevalence in wild boars alone may provide a biased basis for public health concerns; thus, hunters and hunting dogs should always be included in such studies.” (Page 3, Lines 63-66).

1.13. Line 136-137, what domestic animals are referred to in this sentence? because dogs have already been mentioned. Write two separate sentences for the results in pets and humans please.

**Changed:** The bibliography has been added and the sentences have been changed.

**Now you read**:” Furthermore, Brazilian rural areas have higher human and domestic animal seroprevalence of *T. gondii* antibodies than urban areas [23]. In rural areas of northern Brazil, 350/427 (81.3%) local habitants were seropositive to *T. gondii*, mostly associated with cat contact and consumption of wild game meat [24, 25]. On the other hand, a total of 40/189 (21.5%) people living in urban areas at the same state were seropositive to *T. gondii*, associated only to the high stray cat population [24]. In rural areas of southern Brazil, 119/163 (73.0%) owned cats, 159/189 (84.1%) owned dogs, and 227/345 (65.8%) humans were seropositive to *T. gondii* [25]. In urban areas of the same state, 119/729 (16.32%) owned dogs and 248/597 (41.54%) owners were seropositive [19]. (Page 6, Lines 124-134).

1.14. Line 138-139, it is very similar to Line 131. Who says it? Lack of bibliography.

**Changed:** Reviewer is right. Bibliography has been added.

**Now you read:** “The consumption of hunting meat has been considered an emerging risk factor for human infection by *T. gondii* [7]; however, the impact of wild boar hunting on hunters and hunting dogs toxoplasmosis remains to be fully established. Thus, the present study aimed to determine the presence of anti-*Toxoplasma gondii* antibodies in wild boars, hunting dogs, and hunters, and evaluate the associated risk factors for exposure in different areas and biomes of southern and central-western Brazil.” (Page 6, Lines 135-141).

1.15. Line 141, prevalence of antibodies to *T. gondii*. The authors did not detect the infection, they detected the presence of antibodies whose presence does not always coincide with the presence of the pathogen.

**Changed:** Reviewer is right. The sentence has been changed.

**Now you read:**” Thus, the present study aimed to determine the presence of anti-*Toxoplasma gondii* antibodies in wild boars, hunting dogs, and hunters, and evaluate the associated risk factors for exposure in different areas and biomes of southern and central-western Brazil.” (Page 6, 138-141).

1.16. Line 141, the use of Accordingly is not necessary, please deleted.

**Changed:** “Accordingly” has been deleted.

**Now you read:”** Thus, the present study aimed to determine the presence of anti-*Toxoplasma gondii* antibodies in wild boars, hunting dogs, and hunters, and evaluate the associated risk factors for exposure in different areas and biomes of southern and central-western Brazil” (Page 6, 138-141).

1.17. The present cross-sectional study was conducted in the Campos Gerais National Park and periruban areas of Curitiba, Castro, Palmeira, Ponta Grossa, Porto Amazonas, and Teixeira Soares.

Then you can include a description of area: vegetation, climate…

**Changed:** The paragraph has been changed with the description of area.

**Now you read:** “The study was conducted in a natural area of the Campos Gerais National Park and nearby anthropized areas of Campos Gerais region (composed by Curitiba, Castro, Palmeira, Ponta Grossa, Porto Amazonas, and Teixeira Soares municipalities). This southern Brazilian area has a humid temperate climate with an average temperature of 17.5 °C and rainfall index average of 1495 mm^3^. The area is formed by preserved and degraded areas of Atlantic Forest biome, with fields and mixed ombrophilous forests [26]. In addition, samples were collected in the extensive agricultural areas of Aporé municipality, a degraded area of the Cerrado biome in the central-western Brazilian region, which is of tropical climate with average temperature of 23.9°C and rainfall index average of 1539 mm^3^ [27](Fig 1).” (Page 7, Lines 145-156).

1.18. In the Figure1 please delete the word “only” from references. Dog samples, human samples it is ok. But include biomas in the figure title.

The Municipalities: blue, red, violet and light blue areas what kind of biome have? That information is not clear in the Figure.

**Changed:** The word “only” was deleted from references. Locations of sample collections were included in the figure by points and biomes by polygons, thus the localities are differentiated.

1.19. Line 162, serum samples of wild boars, hunting dogs, and hunters were collected. Line 163, ~~during 22 on-field expeditions carried out from~~ between October 2016 and May 2018.

**Changed:** The sentence was changed.

**Now you read:** “Samples of wild boars, hunting dogs, and hunters were conveniently collected between October 2016 to May 2018.” (Page 7, Lines 162-163).

1.20. Line 166, without anti-coagulant and kept at ~~room temperature~~ 25°C.

**Changed:** The sentence has been changed.

**Now you read:** “Samples were placed in tubes without anticoagulant and kept at 25 °C until visible clot retraction. Serum was then separated by centrifugation at 1,500 rpm for five minutes, and stored at -20 °C until processing.” (Page 7-8, Lines 165-168).

1.21. Line 181-188, authors should provide more detail on the performance metrics of the IFAT (sensitivity, specificity).

**Changed:** The sensibility and specificity of IFAT has been added.

**Now you read**:” The described IFAT sensitivity has ranged in different species from 80,4 to 100% and specificity from 91,4% to 95,8% [33].” (Page 8, 188-190).

1.22. The subtitle is “Serum Sample Collection”. Was any other type of sample collected? Change the subtitle to Sample collection.

**Changed:** “Serum Sample Collection” has been changed for “Sample collection”.

**Now you read:** “Sample collection” (Page 7, Line 161).

1.23. Serological diagnosis change to Antibody detection or Serological test.

**Changed: “**Serological diagnosis” has been changed to “Serological test”.

**Now you read:** “Serological test” (Page 8, Line 180).

1.24. Data analysis change to Epidemiological data collection and separate in another subtitle Statistical analysis.

**Changed: “**Data analysis” has been changed for “Epidemiological data collection” and the subtitle “Statistical analysis” has been added.

**Now you read: “**Epidemiological data collection” (Page 8, Line 191).

“Statistical analysis” (Page 9, Line 204).

1.25. Line 191-194, please write separately and what information was collected from the wild boars, dogs and people. Use separate sentences.

**Changed:** The paragraph has been separately rewritten for wild boars, dogs and people.

**Now you read: “**Epidemiological analyses were performed based on a questionnaire associated with wild boar exposure to *T. gondii* which included age, sex, sample location and free-range or captured wild boar (S1 Dataset).

Epidemiological analyses were performed based on a questionnaire associated with hunting dog exposure to *T. gondii* which included age, body size, sex, hunting experience, hunting practices, hunting meat consumption, current wild boar hunting, dog mobility, feeding of dog food and leftover (S1 Dataset).

Epidemiological analyses were performed based on a questionnaire associated with hunter exposure to *T. gondii* which included age, sex, household location, hunting practices, hunting meat consumption, occupation, number of minimum wages, school level, washing fruits and vegetables and hand contact with earth and sand (S1 Dataset).” (Page 9, Lines 192-203).

1.26. Line 199, *T. gondii*

**Changed:** *T. gondii* has been italicized throughout the manuscript.

**Now you read:** “Fisher’s exact test was used to determine the bivariate association between the studied variables, and odds ratios (OR) were used for the association of *T. gondii* prevalence to potential risk factors.” (Page 9, Line 207-209).

1.27. Line 158, (Fig 1) and Line 208 (Figure 1) ??

**Changed:** (Figure 1) has been changed for (Fig 1).

**Now you read:** “A map illustrating the municipalities of sampling (source: free access Brazilian databases <ftp://geoftp.ibge.gov.br/organizacao_do_territorio/malhas_territoriais/malhas_municipais/municipio_2015/>) and biomes of the studied regions (source: free access Brazilian databases <https://downloads.ibge.gov.br/downloads_geociencias.htm>) was produced by authors, using these free open access shapefiles and performed on GIS software using ARCGIS 10 [35] and presented (Fig 1).” (Page 10, Line 215-220).

1.28. Line 206, correct please bioma for biome in all the manuscripts and the figure.

**Changed:** “Bioma” has been replaced by “biomes”.

**Now you read:** “A map illustrating the municipalities of sampling (source: free access Brazilian databases <ftp://geoftp.ibge.gov.br/organizacao_do_territorio/malhas_territoriais/malhas_municipais/municipio_2015/>) and biomes of the studied regions (source: free access Brazilian databases <https://downloads.ibge.gov.br/downloads_geociencias.htm>) was produced by authors, using these free open access shapefiles and performed on GIS software using ARCGIS 10 [35] and presented (Fig 1). (Page 10, Line 215-220).

1.29. Line 225, antibodies to *T. gondii* were detected in 15/71 (21.1% - CI 95% 12.7-32.6%) wild boars, 49/157 (31.2% -226 CI 95% 24.8-38.3%) hunting dogs and 15/49 (32.7% - CI 95% 21.7-47.1%) 227 hunters.

**Changed:** Sentence has been rewritten.

**Now you read:** “Antibodies to *T. gondii* were detected in 15/71 (21.1%, CI 95% 12.7-32.6%) wild boars, 49/157 (31.2%, CI 95% 24.8-38.3%) hunting dogs, and 15/49 (32.7%, CI 95% 21.7-47.1%) hunters.” (Page 10, Lines 235-237).

1.30. Line 227*, T. gondii*

**Changed:** The original sentence has been changed for “Antibodies to *T. gondii* were detected in 15/71 (21.1% - CI 95% 12.7-32.6%) wild boars, 49/157 (31.2% -226 CI 95% 24.8-38.3%) hunting dogs and 15/49 (32.7% - CI 95% 21.7-47.1%) 227 hunters.”

**Now you read:** “Antibodies to *T. gondii* were detected in 15/71 (21.1%, CI 95% 12.7-32.6%) wild boars, 49/157 (31.2%, CI 95% 24.8-38.3%) hunting dogs, and 15/49 (32.7%, CI 95% 21.7-47.1%) hunters.” (Page 10, Lines 235-237).

1.31. Line 234, “Seropositivity for *T. gondii* was statistically higher in 12/14 (85.7%) 235 captured wild boars when compared to 5/57 (7.0%) free-range wild boars”. What the difference between captured and free-range wild boars?? explain it in Sample collection please.

**Changed:** Reviewer is right, and the text has been corrected to better explain the difference between captured and free-range wild boars. The term “free-range wild boar” was used for wild boars sampled following slaughter by firearm in natural, agricultural and anthropized areas. The term “captured wild boars” was used for wild boars which were captured as piglets and kept in local farms of anthropized areas at southern Brazil, also sampled following sedation and physical restrain.

**Now you read:** “Free-range wild boars from agricultural and anthropized areas were sampled following slaughter by firearm, under the Brazilian hunting laws for invasive exotic species, with legally registered hunters and correspondent hunting dogs at the Brazilian Institute of the Environment and Renewable Natural Resources (IBAMA Normative Instruction 03/2013). In addition, free-range wild boars from a natural area in the Vila Velha State Park were baited, photo-monitored, trapped and slaughtered by firearm, following previous authorization by the Brazilian Environmental Biodiversity System (SISBIO license 61805-2/2017). Finally, previously captured free-range piglets, kept and raised at two local farms of anthropized areas in southern Brazil, considered as captured wild boars, were also sampled following sedation and physical restraint.” (Page 8, Lines 169-179).

1.32. Line 243, change “hunting dogs were not statistically significant between males and females (p=0.135)”

**Changed:** The sentence has been changed.

**Now you read:** “Associated risk factors for hunting dogs were not statistically significant between males and females (p=0.135);” (Page 15, Lines 253-254).

1.33. Line 244, please deleted gender and use sex.

**Changed:** “Gender” has been changed for “sex”.

**Now you read: “**Associated risk factors for hunting dogs were not statistically significant between males and females (p=0.135);” (Page 15, Lines 253-254).

1.34. Line 258, please start the paragraph “The statistical analysis showed …” for a correct wording in English.

**Changed:** The paragraph has been changed.

**Now you read: “**The statistical analysis for risk factors in hunters showed no significant association for occupation, between being retired or a student to private (p=0.464) or public (p=0.129) workers;” (Page 15, Line 269-271).

1.35. Line 268, meat (p=0.024) and raw fish (p=0.026) were statistically 268 significant and protective.

**Changed:** The term “protective” has been deleted.

**Now you read: “**Consumption of uncooked meat (p=0.024), raw meat (p=0.024), and raw fish (p=0.026) were statistically significant (Table 1).” (Page 16, Lines 278-280).

“As expected, human consumption of uncooked meat (p=0.024), raw meat (p=0.024), and raw fish (p=0.026) was associated with increased seropositivity, probably due to the consumption of uninspected meat sources.” (Page 19, 367-370).

1.36. Line 272, “To the authors knowledge, the present study has been the first concomitant report of wild boar, hunting dog, and hunter”. The authors must make an adequate review for the work, it cannot be simply an appreciation. You can write: This is the first study that reveals the presence of antibody to *T. gondii* in wild boars, hunting dogs and hunters and carries out an epidemiological risk analysis.

**Changed:** Reviewer is right and the paragraph has been corrected.

**Now you read:** “To the authors' knowledge, this is the first study reporting the presence of antibodies to *T. gondii* in wild boars, hunting dogs, and hunters, and carries out an epidemiological risk analysis.” (Page 16, Lines 282-284).

1.37. “performed herein in southern and central-western regions of Brazil”. Is the first study in Brazil? in that region of Brazil?

**Changed:** This is the first study that reveals the concomitant presence of antibody to *T. gondii* in wild boars, hunting dogs and hunters around the world. Despite this, previous studies with wild boars and *T. gondii* have been conducted in southern and central-western of Brazil, such as:

Santos LMJF, Farias NAR, Oliveira PA, Cademartori BG, Ramos TS, Oliveira FC, et al. Presence of Toxoplasma gondii infection in wild boar in southern Brazil. Sch J Agric Vet Sci. 2016; 3(3): 238-241

Brandão LNS, Rosa JMA, Kramer B, Sousa ATHI, Trevisol IM, Nakazato L, et al. Detection of Toxoplasma gondii infection in feral wild boars (Sus scrofa) through indirect hemagglutination and PCR. Ciênc Rural. 2019;49(3): e20180640. http://dx.doi.org/10.1590/0103-8478cr20180640.

The sentence has been changed.

**Now you read:** “To the authors' knowledge, this is the first study reporting the presence of antibodies to *T. gondii* in wild boars, hunting dogs, and hunters, and carries out an epidemiological risk analysis.” (Page 16, Lines 282-284).

1.38. Line 276, the seroprevalence obtained to *T. gondii* antibodies in the present study is higher than….

**Changed:** The sentence has been changed.

**Now you read: “**The seroprevalence of *T. gondii* antibodies in the present study is higher than in (…)” (Page 16, Lines 291-292).

1.39. Line 277-282, how is the comparison with studies in the South America? Could wild boar with antibodies to *T. gondii* expand to other countries that have not positive to *T. gondii* wild boar?

**Changed:** Only a single other study has been performed with wild boars and *Toxoplasma gondii* in South American countries.

**Now you read:** “In South America, the seroprevalence in wild boars herein was higher than the reported in the only comparable study in Argentina [8].” (Page 16, 298-299).

1.40. Line 328, please deleted herein in all de manuscript.

**Changed:** “Herein” has been deleted throughout the manuscript.

**Now you read: “**Seroprevalence of toxoplasmosis in Brazilian wild boars was within the national and international range including South and North Americas, Europe and Asia, posting wild boars as potential environmental sentinels for *T. gondii* presence.” (Page 2, Lines 45-47).

“The present study has consistently corroborated to such findings, where the peridomestic environment and the diet of owned dogs and owners (associated to domestic cat proximity) suggest increased exposure to *T. gondii* when compared to hunting dogs and hunters.” (Page 17, 311-315).

“Despite the low seropositivity for *T. gondii* found in wild boars in the present study, a cumulative pattern due to the overtime consumption of raw wild boar meat could have impacted the seropositivity of dogs and hunters.” (Page 21, 410-412).

1.41. The authors should discuss why they used MAT for wild boars and IFAT for hunting dogs and hunters.

**Changed:** Reviewer is right. The use of serological tests for each species has been discussed.

**Now you read:** “Different serological tests were used among wild boars, dogs, and human samples due to the requirement of species-specific conjugates to perform IFAT, which is not commercially available for wild animals. Thus, the serological status of wild boars was assessed by MAT. Also, both MAT and IFAT have shown adequate performance to detect IgG antibodies in pigs, with similar specificity and sensitivity [35].” (Page 16, Lines 285-290).

1.42. In the discussion they should highlight in cases of significant statistical differences and discuss what they should be.

**Changed:** News sentences have been added to discussion.

**Now you read:** “In the present study, consumption of raw rat meat had significantly increased the risk of dog seropositivity, which is in agreement with a previous seroprevalence study, where dog contact with rats was associated with a higher risk of *Toxoplasma* infection [19]. Although the dog’s habit of hunting increased the risk of dog exposure; no previous study has focused on wild boars as an associated risk for toxoplasmosis in dogs. Since consumption of raw wild boar meat has not been associated with dog seropositivity, exposure may be due to outdoors activity, leading to access to other infection sources.”. (Page 18, 344-351).

“As previously shown in a systematic review, sanitary inspection and pig farming systematization have been crucial to decrease *T. gondii* occurrence, and consumption of organically farmed pigs results in significantly higher prevalence than consuming meat from conventional or small farms [48].” (Page 19, Lines 370-374).

“Such slightly higher seropositivity in hunting dogs is in agreement with previous studies in rural areas of southern Brazil, probably as a consequence of rat ingestion. This study proposed the use of dogs as potential environmental sentinels since dogs and humans were sharing infection sources [25].” (Page 19, Lines 363-367).

1.43. Why the title of the hunting dogs is higher? to what could it be?

**Changed:** Dogs may be environmental sentinels to *Toxoplasma gondii*. In this context, probably due to rat ingestion.

**Now you read:** “Such slightly higher seropositivity in hunting dogs is in agreement with previous studies in rural areas of southern Brazil, probably as a consequence of rat ingestion. This study proposed the use of dogs as potential environmental sentinels since dogs and humans were sharing infection sources [25].” (Page 19, Lines 363-367).

1.44. The authors could discuss about wild boars feeding habits.

**Changed:** The relationship between wild boars feed habits and *T. gondii* exposure has been discussed.

**Now you read:** “Due to opportunistic and omnivorous habits, wild boars may have several sources for *T. gondii* exposure [49,50]. Not surprisingly, a comparative study has shown a higher prevalence of *T. gondii* infection in omnivores (17/105, 16.2% in wild boars) and carnivores (15/94, 15.9% in red foxes), when compared to herbivores (3/121, 2.48% in roe deer), suggesting the importance of tissue cysts for transmission [51].” (Page 20, Lines 377-383).

1.45. Line 345-351, I do not agree that in the last paragraph there is reference to another pathogen that is not the one that motivates this investigation. Please deleted or rewrite.

**Changed:** The reviewer is right. The last paragraph has been deleted.

**Now you read:** “Future studies are needed to fully establish body load and distribution of naturally infected, free-range wild boars in different environmental settings.” (Page 20, Lines 404-406).

1.46. Line 356-357, this sentence is inconsistent. You cannot make that claim with 71 wild boar and 157 hunting dog serum samples.

**Changed:** Reviewer is right. The sentence has been changed with our sample size limitations.

**Now you read:** “Despite the limited sample size available in the present study, our findings have comparatively shown that wild boars may be less exposed to infection than hunting dogs and hunters in both Brazilian regions.” (Page 21, 408-410).

1.47. Line 358-362, this sentence is inconsistent. You cannot make that claim. Wild boars are able to travel kilometers in just one night.

**Changed:** The sentence has been changed and bibliography has been added.

**Now you read:** “Despite that 14/20 (70.0%) trapped wild boars in the state park were female, the only positive sample was from a male wild boar. Although wild boars may be able to travel long distances overnight, a much higher variation of the home range has been reported in males when compared to females [42; 43]. Moreover, since in-park hunting is prohibited [5, 6], the higher frequency of female trapping may be due to a larger population, as females may be less likely to cross park limits and be hunted. In addition, wild boar activity and roaming distance may vary, including diurnal or nocturnal preferences, which is mostly related to human proximity [43], making the natural areas an ideal nursery habitat for females and their piglets. Therefore, it is reasonable to speculate that female wild boars sampled in-park may travel shorter distances and are likely less exposed to *Toxoplasma gondii*.” (Page 17-18, Lines 324-335).

**Reviewer 1** Final comments

The research and the results obtained are relevant because published works of this subject in South America are reduced, while wild boar population continues to grow and expand by the continent.

**Answer:** We appreciate the positive comment on the study.

- The manuscript needs editing for English.

**Answer:** The English-Language review has been performed.

- There are also some statements that are not clear as written.

- The authors should deepen the discussion.

The study is important to be published with the pertinent corrections.

Reviewer #2 (purple intext):

1. Is the manuscript technically sound, and do the data support the conclusions?

Reviewer #2: Partly

2. Has the statistical analysis been performed appropriately and rigorously?

Reviewer #2: Yes

3. Have the authors made all data underlying the findings in their manuscript fully available?

Reviewer #2: Yes

4. Is the manuscript presented in an intelligible fashion and written in standard English?

Reviewer #2: Yes

Review Comments to the Author

2.1. The article is quite well written. However, provided data do not justify very long descrip-tion and most of statistical analyses.

**Changed:** Reviewer is right. An explanation has been added to intext.

**Now you read:** “One limitation of our study is the low number of samples, which generated insufficient data to provide the basis for a representative statistical description and analyses. However, there is a lack of studies involving hunters and hunting dogs, probably due to difficulties in accessing the population and their refusal to participate in the study. Additionally, essential data related to population ratios, animal locations, and epidemiology is challenging to obtain in wildlife settings [37]. Thus, as the first description of three populations altogether (wild boars, hunting dogs, and hunters), the statistical description in the present study is essential to develop new hypotheses and discussions, encouraging further, more comprehensive studies.” (Page 20-21, Lines 395-404).

2.2. For example, text in lines from 96 to 121 can be written in much shorter form. Moreover, there is many repetitions, for the same information provided in different words.

**Changed:** Reviewer is right. The paragraph has been shorted.

**Now you read:** “Seroprevalence of *T. gondii* has been extensively studied in free-range wild boars throughout the world [7]. In South America, Argentina has recently reported the presence of antibodies to *T. gondii* in 18/144 (12.5%) free-range wild boars [8], whereas, in Brazil, the positivity reported was 14/306 (4.5%) in young farmed animals and 5/34 (14.28%) in free-range wild boars [9]. Another study in Brazil reported the seropositivity of 0/7 (0.0%), 16/101 (15.8%), and 3/14 (21.4%) in free-range wild boars from different regions [10], corroborating with the worldwide in-country variation on *T. gondii* exposure. In European wild boars, the seroprevalence of *T. gondii* ranges from 10/150 (6.7%) in Switzerland to 8/8 (100%) in Portugal [11, 12]. In Asia, the reported ranges are from 1/90 (1.1%) in Japan to 152/426 (35.6%) in South Korea [13,14]. Lastly, in North America, seropositivity has been reported from 34/376 (9.0%) to 181/227 (49.0%) in free-range wild boars from the United States [15,16].“ (Page 5, 96-108).

2.3. Article describe the first seroprevalence study in wild boars, hunted dogs and hunters. However, presented text is not good enough for publication. For example small groups and not representative sample size are not good enough for risk factor analysis for hunter (lines 258- 269). Game might be a source of parasite for dogs and hunters but contact with cats, soil or raw beef too.

**Changed:** Reviewer is right. An explanation about statistical limitations has been added to intext. In addition, all necessary parameters for ideal calculations (population ratios, use animal areas, preliminary data about epidemiological studies) may be difficult to obtain in wildlife studies. A reference study about such aspects have presented options for these calculations in wildlife populations (Czaplewski et al, 1983).

**Now you read:** “One limitation of our study is the low number of samples, which generated insufficient data to provide the basis for a representative statistical description and analyses. However, there is a lack of studies involving hunters and hunting dogs, probably due to difficulties in accessing the population and their refusal to participate in the study. Additionally, essential data related to population ratios, animal locations, and epidemiology is challenging to obtain in wildlife settings [37]. Thus, as the first description of three populations altogether (wild boars, hunting dogs, and hunters), the statistical description in the present study is essential to develop new hypotheses and discussions, encouraging further, more comprehensive studies.” (Page 20-21, Lines 395-404).

2.4. The list of references is too long and some cited articles in text are missing for example:

Witkowski L, Czopowicz M, Nagy DA, Potarniche AV, Aoanei MA, Imomov N, Mickiewicz M, Welz M, Szaluś-Jordanow O, Kaba J. Seroprevalence of Toxoplasma gondii in wild boars, red deer and roe deer in Poland. Parasite. 2015;22:17. doi: 10.1051/parasite/2015017.

**Changed:** The references have been checked and completed.

2.5. In my opinion article should be shortened and in very comprehensive form published in an-other journal.

**Changed:** An explanation about study limitations has been added to intext.

**Now you read:** “One limitation of our study is the low number of samples, which generated insufficient data to provide the basis for a representative statistical description and analyses. However, there is a lack of studies involving hunters and hunting dogs, probably due to difficulties in accessing the population and their refusal to participate in the study. Additionally, essential data related to population ratios, animal locations, and epidemiology is challenging to obtain in wildlife settings [37]. Thus, as the first description of three populations altogether (wild boars, hunting dogs, and hunters), the statistical description in the present study is essential to develop new hypotheses and discussions, encouraging further, more comprehensive studies.” (Page 20-21, Lines 395-404).

Dear Dr. Homewood

,

Please find attached our reviewed manuscript entitled "*Seroprevalence of anti-Toxoplasma gondii antibodies in wild boars (Sus scrofa), hunting dogs, and hunters of Brazil "* (PONE-D-19-16414). All the corrections indicated were fully accepted, addressed and rewritten.

Thank you for the opportunity and please don’t hesitate to contact us with any further question.

Sincerely,

Alexander Welker Biondo, DMV, MSc, PhD.

Professor, Department of Veterinary Medicine, UFPR, Brazil

Visiting Professor, Purdue University, IN, USA

E-mail: [abiondo@ufpr.br](mailto:abiondo@ufpr.br) / Phone: +55 (41) 3350-5812

1.Thank you for updating your data availability statement. You note that your data are available within the Supporting Information files, but no such files have been included with your submission. At this time we ask that you please upload your minimal data set as a Supporting Information file, or to a public repository such as Figshare or Dryad.

Please also ensure that when you upload your file you include separate captions for your supplementary files at the end of your manuscript.

As soon as you confirm the location of the data underlying your findings, we will be able to proceed with the review of your submission.

**Changed:** The supporting information has been added.

**Now you read: “**Epidemiological analyses were performed based on a questionnaire associated with wild boar exposure to *T. gondii* which included age, sex, sample location and free-range or captured wild boar (S1 Dataset).

Epidemiological analyses were performed based on a questionnaire associated with hunting dog exposure to *T. gondii* which included age, body size, sex, hunting experience, hunting practices, hunting meat consumption, current wild boar hunting, dog mobility, feeding of dog food and leftover (S1 Dataset).

Epidemiological analyses were performed based on a questionnaire associated with hunter exposure to *T. gondii* which included age, sex, household location, hunting practices, hunting meat consumption, occupation, number of minimum wages, school level, washing fruits and vegetables and hand contact with earth and sand (S1 Dataset).” (Page 9, Lines 192-203).

**“**S1 Dataset. Data source and results of serological tests to *Toxoplasma gondii* in wild boars, hunting dogs and hunters.” (Page 21, Lines 418-419).

2. We note that Figure 1 in your submission contains a map image which may be copyrighted.

All PLOS content is published under the Creative Commons Attribution License (CC BY 4.0), which means that the manuscript, images, and Supporting Information files will be freely available online, and any third party is permitted to access, download, copy, distribute, and use these materials in any way, even commercially, with proper attribution. For these reasons, we cannot publish previously copyrighted maps or satellite images created using proprietary data, such as Google software (Google Maps, Street View, and Earth). For more information, see our copyright guidelines: http://journals.plos.org/plosone/s/licenses-and-copyright.

We require you to either (a) present written permission from the copyright holder to publish these figures specifically under the CC BY 4.0 license, or (b) remove the figures from your submission:

a.You may seek permission from the original copyright holder of Figure 1 to publish the content specifically under the CC BY 4.0 license.

We recommend that you contact the original copyright holder with the Content Permission Form (http://journals.plos.org/plosone/s/file?id=7c09/content-permission-form.pdf) and the following text:

b. If you are unable to obtain permission from the original copyright holder to publish these figures under the CC BY 4.0 license or if the copyright holder’s requirements are incompatible with the CC BY 4.0 license, please either i) remove the figure or ii) supply a replacement figure that complies with the CC BY 4.0 license. Please check copyright information on all replacement figures and update the figure caption with source information. If applicable, please specify in the figure caption text when a figure is similar but not identical to the original image and is therefore for illustrative purposes only.

**Changed:** The map has been produced by our own team, using free open access shapefiles described in “Statistical analysis” (source: free access Brazilian databases <ftp://geoftp.ibge.gov.br/organizacao_do_territorio/malhas_territoriais/malhas_municipais/municipio_2015/> and biomes of the studied regions; source: free access Brazilian databases https://downloads.ibge.gov.br/downloads_geociencias.htm) and performed on GIS software. For this reason, we understand that there is no need to have a specific license to use the figure.

**Now you read: “**A map illustrating the municipalities of sampling (source: free access Brazilian databases ftp://geoftp.ibge.gov.br/organizacao_do_territorio/malhas_territoriais/malhas_municipais/municipio_2015/) and biomes of the studied regions (source: free access Brazilian databases https://downloads.ibge.gov.br/downloads_geociencias.htm) was produced by authors, using these free open access shapefiles and performed on GIS software using ARCGIS 10 [35] and presented (Fig 1).” (Pages 10, 215-220).

“**Fig 1.** Sampling locations of wild boars, hunting dogs, and hunters from southern and central-western Brazil. The map has been produced by authors, using free open access shapefiles described in methodology section and performed on GIS software using ARCGIS 10 [35].” (Page 7, Lines 158-161).
