# Supplementary material for: Seroprevalence of anti-Toxoplasma gondii antibodies in wild boars (Sus scrofa), hunting dogs, and hunters of Brazil
Source: PLoS One. 2019 Oct 11;14(10):e0223474. doi: 10.1371/journal.pone.0223474 (PMC6788692; doi:10.1371/journal.pone.0223474)
Supplement: Attachment — Submitted filename: Response to reviewers 11thSept2019.docx [file pone.0223474.s004.docx]

Curitiba, Brazil, September 11^th^, 2019

**Response to reviewers**

To: Paulo Lee Ho, PhD / Academic Editor - PLOS ONE

Re: author’s PLOS ONE manuscript resubmission (PONE-D-19-16414)

Dear Dr. Ho,

Please find attached our reviewed manuscript entitled " Seroprevalence of anti-*Toxoplasma gondii* antibodies in wild boars (*Sus scrofa*), hunting dogs, and hunters of Brazil " (PONE-D-19-16414). All the corrections and suggestions indicated by the reviewers were fully accepted, addressed and rewritten.

A letter has been written to pinpoint the reviewers’ requests, with a detailed list of corrections and a description of changes made within the manuscript. To better identify the corrections of each reviewer, changes were highlighted in different colors in the revised manuscript.

Thank you for the opportunity and please don’t hesitate to contact us with any further question.

Sincerely,

Alexander Welker Biondo, DMV, MSc, PhD.

Professor, Department of Veterinary Medicine, UFPR, Brazil

Visiting Professor, Purdue University, IN, USA

E-mail: [abiondo@ufpr.br](mailto:abiondo@ufpr.br) / Phone: +55 (41) 3350-5812

**Manuscript PONE-D-19-16414**

**Title:** "Seroprevalence of anti-*Toxoplasma gondii* antibodies in wild boars (*Sus scrofa*), hunting dogs, and hunters of Brazil."

**Dear Reviewer #1,**

Firstly, we appreciate all your suggestions and comments and our manuscript. Certainly, your suggestions were very important to improve the manuscript.

**1. Reviewer #1 (green intext):**

Comments to the Author

1. If the authors have adequately addressed your comments raised in a previous round of review and you feel that this manuscript is now acceptable for publication, you may indicate that here to bypass the “Comments to the Author” section, enter your conflict of interest statement in the “Confidential to Editor” section, and submit your "Accept" recommendation.

Reviewer #1: (No Response)

2. Is the manuscript technically sound, and do the data support the conclusions?

The manuscript must describe a technically sound piece of scientific research with data that supports the conclusions. Experiments must have been conducted rigorously, with appropriate controls, replication, and sample sizes. The conclusions must be drawn appropriately based on the data presented.

Reviewer #1: Yes

3. Has the statistical analysis been performed appropriately and rigorously?

Reviewer #1: Yes

4. Have the authors made all data underlying the findings in their manuscript fully available?

The PLOS Data policy requires authors to make all data underlying the findings described in their manuscript fully available without restriction, with rare exception (please refer to the Data Availability Statement in the manuscript PDF file). The data should be provided as part of the manuscript or its supporting information, or deposited to a public repository. For example, in addition to summary statistics, the data points behind means, medians and variance measures should be available. If there are restrictions on publicly sharing data—e.g. participant privacy or use of data from a third party—those must be specified.

Reviewer #1: Yes

5. Is the manuscript presented in an intelligible fashion and written in standard English?

PLOS ONE does not copyedit accepted manuscripts, so the language in submitted articles must be clear, correct, and unambiguous. Any typographical or grammatical errors should be corrected at revision, so please note any specific errors here.

Reviewer #1: Yes

**Review Comments to the Author**

**1.1.** The abstract is too long and it can be improved

**Changed:** The abstract has been shortened and improved.

**Now you read:** “Seroprevalence of *Toxoplasma gondii* has been extensively studied in wild boars worldwide due to the emerging risk for human infection through meat consumption. However, this is the first study that reports toxoplasmosis seroprevalence in wild boars, wild boar hunters and their hunting dogs. The aim of the present study was to evaluate the seroprevalence of anti-*T. gondii* antibodies in the complex wild boars, hunting dogs and hunters, and to determine the risk factors associated with seropositivity in southern and central-western Brazil. Overall, anti-*T. gondii* seropositivity was observed in 15/71 (21.1%) wild boars by modified agglutination test (MAT); and 49/157 (31.2%) hunting dogs and 15/49 (32.7%) hunters by indirect immunofluorescent antibody test (IFAT). Seroprevalence of toxoplasmosis in Brazilian wild boars was within the national and international range, posting wild boars as potential environmental sentinels for *T. gondii* presence. In addition, the findings have comparatively shown that wild boars have been less exposed to infection than hunting dogs or hunters in both Brazilian regions. Seropositivity for *T. gondii* was statistically higher in 12/14 (85.7%) captured wild boars when compared to 5/57 (7.0%) free-range wild boars (p=0.000001). Similarly, captured wild boars from anthropized areas were more likely to be seropositive than of natural regions (p=0.000255). When in multiple regression model, dogs with the habit of wild boar hunting had significant more chance to be positive (adjusted-OR 4.62 CI 95% 1.16-18.42). Despite potential as sentinels of environmental toxoplasmosis, seroprevalence in wild boars alone may provide a biased basis for public health concerns; thus, hunters and hunting dogs should be always be included in such studies. Although hunters should be aware of potential *T. gondii* infection, wild boars from natural and agricultural areas may present lower protozoa load when compared to wild boars from anthropized areas, likely by the higher presence of domestic cats as definitive hosts.” (Page 2-3, Lines 35-61).

**1.2.** Line 37, Surprisingly is not a proper word.

However, this is the first study that reports toxoplasmosis seroprevalence in wild boars, wild boar hunters and their hunting dogs.

**Changed:** Reviewer is right. “Surprisingly” has been changed to “however”.

**Now you read:** “However, this is the first study that reports toxoplasmosis seroprevalence in wild boars, wild boar hunters and their hunting dogs.” (Page 2, Lines 37-38).

**1.3.** Line 39, The aim of the present study was to evaluate the seroprevalence of anti-*T. gondii* antibodies in the complex wild boars, hunting dogs, and hunters, and to determine the risk factors associated with seropositivity in southern and central-western Brazil.

Consider that the abstract should incorporate all subtitles of the manuscript in an adequate and balanced way

**Changed:** The sentence has been changed.

**Now you read:** “The aim of the present study was to determine the presence of anti-*Toxoplasma gondii* antibodies in wild boars, hunting dogs, and hunters, and evaluate the associated risk factors for exposure in different areas and biomes of southern and central-western Brazil.” (Page 2, Lines 38-42).

“Seroprevalence of *Toxoplasma gondii* has been extensively studied in wild boars worldwide due to the emerging risk for human infection through meat consumption. However, this is the first study that reports toxoplasmosis seroprevalence in wild boars, wild boar hunters and their hunting dogs. The aim of the present study was to evaluate the seroprevalence of anti-*T. gondii* antibodies in the complex wild boars, hunting dogs and hunters, and to determine the risk factors associated with seropositivity in southern and central-western Brazil. Overall, anti-*T. gondii* seropositivity was observed in 15/71 (21.1%) wild boars by modified agglutination test (MAT); and 49/157 (31.2%) hunting dogs and 15/49 (32.7%) hunters by indirect immunofluorescent antibody test (IFAT). Seroprevalence of toxoplasmosis in Brazilian wild boars was within the national and international range, posting wild boars as potential environmental sentinels for *T. gondii* presence. In addition, the findings have comparatively shown that wild boars have been less exposed to infection than hunting dogs or hunters in both Brazilian regions. Seropositivity for *T. gondii* was statistically higher in 12/14 (85.7%) captured wild boars when compared to 5/57 (7.0%) free-range wild boars (p=0.000001). Similarly, captured wild boars from anthropized areas were more likely to be seropositive than of natural regions (p=0.000255). When in multiple regression model, dogs with the habit of wild boar hunting had significant more chance to be positive (adjusted-OR 4.62 CI 95% 1.16-18.42). Despite potential as sentinels of environmental toxoplasmosis, seroprevalence in wild boars alone may provide a biased basis for public health concerns; thus, hunters and hunting dogs should be always be included in such studies. Although hunters should be aware of potential *T. gondii* infection, wild boars from natural and agricultural areas may present lower protozoa load when compared to wild boars from anthropized areas, likely by the higher presence of domestic cats as definitive hosts.” (Page 2-3, Lines 35-61).

1.4. Line 86, Felidae no italic please

Changed: The italicized world has been corrected.

**Now you read:** **“***Toxoplasma gondii* is a coccidian parasite relying on cats and other Felidae as definitive hosts, which may shed fecal oocysts that can infect a variety of intermediate hosts (avian and mammal species). [1, 2].” (Page 4, Lines 76-78).

1.5. Line 86-87, which may shed fecal oocysts that can infect a variety of intermediate hosts (avian and mammal species).

**Changed:** The intermediate hosts have been added to the sentence.

**Now you read: “***Toxoplasma gondii* is a coccidian parasite relying on cats and other Felidae as definitive hosts, which may shed fecal oocysts that can infect a variety of intermediate hosts (avian and mammal species) [1, 2].” (Page 4, Lines 76-78).

1.6. Line 89, ingestion of infected raw or undercooked meat

**Changed:** “Contaminated” has been replaced by “infected”.

**Now you read**: “Since infected intermediate hosts may harbor viable tissue cysts for years, human beings may be infected by ingestion of infected raw or undercooked meat [2, 3].” (Page 4, Lines 78-80).

1.7. Line 92, production. Delete please. Write only livestock.

**Changed:** “Livestock” has been kept and “production” deleted.

**Now you read: “**Its presence produces a substantial negative impact on health, livestock, and native wildlife [5].” (Page 4, Lines 82-83).

1.8. Line 95, “Thus, dogs could also directly exposed to *T. gondii* infection.” Sentence is not necessary at this point in the manuscript.

**Changed:** The sentence has been deleted.

1.9. Line 135, “The consumption of hunting meat has been considered an emerging risk factor for human infection by T. gondii [7]; however, the impact of wild boar hunting on hunters and hunting dogs toxoplasmosis remains to be fully established. Thus,”

Please delete this paragraph….The aim of the present study was ……

**Changed:** The sentences have been deleted.

**Now you read:** “The aim of the present study was to determine the presence of anti-*Toxoplasma gondii* antibodies in wild boars, hunting dogs, and hunters, and evaluate the associated risk factors for exposure in different areas and biomes of southern and central-western Brazil.” (Page 6, Lines 125-128).

1.10. Study area: In Figure 1 you use Atlantic forest, Cerrado and Degraded area: is the degraded area the same as degraded areas of Atlantic Forest biome? and Atlantic forest the same as preserved of Atlantic forest?? Please use the same words in the figure and in the text.

**Changed:** The reviewer is right. Figure and text have been changed to better describe such areas. “Degraded areas” was replaced by “agricultural and anthropized areas (both biomes)” in the figure and explained in the text as “although anthropized and agricultural areas in both biomes have been considered as degraded areas, such areas were considered separately for statistical analyses.”

**Now you read:** “The study was conducted in a natural area of the Campos Gerais National Park, nearby anthropized areas of Campos Gerais region (composed by Curitiba, Castro, Palmeira, Ponta Grossa, Porto Amazonas, and Teixeira Soares municipalities) in southern Brazil and in an agricultural area at Aporé municipality of central-western Brazil. This southern Brazilian area has a humid temperate climate with an average temperature of 17.5 °C and rainfall index average of 1495 mm^3^. The area is formed by natural and degraded areas of Atlantic Forest biome, with fields and mixed ombrophilous forests [26]. The extensive agricultural area of Aporé municipality is a degraded area of the Cerrado biome in the central-western Brazilian region, which is of tropical climate with average temperature of 23.9°C and rainfall index average of 1539 mm^3^ [27]. Although anthropized and agricultural areas in both biomes have been considered as degraded areas, such areas were considered separately for statistical analyses (Fig 1).” (Page 6, Lines 132-145).

1.11. Line 152, “In addition, samples were collected in”… Please delete this sentence. “The extensive….

**Changed:** The sentence has been deleted.

**Now you read:** “The study was conducted in a natural area of the Campos Gerais National Park, nearby anthropized areas of Campos Gerais region (composed by Curitiba, Castro, Palmeira, Ponta Grossa, Porto Amazonas, and Teixeira Soares municipalities) in southern Brazil and in an agricultural area at Aporé municipality of central-western Brazil. This southern Brazilian area has a humid temperate climate with an average temperature of 17.5 °C and rainfall index average of 1495 mm^3^. The area is formed by natural and degraded areas of Atlantic Forest biome, with fields and mixed ombrophilous forests [26]. The extensive agricultural area of Aporé municipality is a degraded area of the Cerrado biome in the central-western Brazilian region, which is of tropical climate with average temperature of 23.9°C and rainfall index average of 1539 mm^3^ [27]. Although anthropized and agricultural areas in both biomes have been considered as degraded areas, such areas were considered separately for statistical analyses (Fig 1).” (Page 6, Lines 132-145).

1.12. Sample collections

Change the order of the two paragraphs. First how were the catches, and then how was the sample extraction.

**Changed:** The order of the paragraphs has been changed.

**Now you read: “**Free-range wild boars from agricultural and anthropized areas were sampled following slaughter by firearm, under the Brazilian hunting laws for invasive exotic species, with legally registered hunters and correspondent hunting dogs at the Brazilian Institute of the Environment and Renewable Natural Resources (IBAMA Normative Instruction 03/2013). In addition, free-range wild boars from a natural area in the Vila Velha State Park were baited, photo-monitored, trapped and slaughtered by firearm, following previous authorization by the Brazilian Environmental Biodiversity System (SISBIO license 61805-2/2017). Finally, previously captured free-range piglets, kept and raised at two local farms of anthropized areas in southern Brazil, considered as captured wild boars, were also sampled following sedation and physical restraint.

Samples of wild boars, hunting dogs, and hunters were conveniently collected between October 2016 to May 2018. Blood collection was performed by intracardiac puncture immediately after death in wild boars, by jugular puncture in dogs, and by cephalic puncture in hunters. Samples were placed in tubes without anticoagulant and kept at 25 °C until visible clot retraction. Serum was then separated by centrifugation at 1,500 rpm for five minutes, and stored at -20 °C until processing.” (Page 6-7, Lines 153-170).

1.13. Statistical analysis

This sentence should go to the beginning of the paragraph: Statistical analysis was performed using SPSS 20.0 215 [34].

**Changed:** Now, the sentence starts the paragraph.

**Now you read: “**Statistical analysis was performed using SPSS 20.0 [34].” (Page 9, Line 196).

1.14. Line 284, “To the authors' knowledge”. Please delete this phrase.

This is the first study that reposts the presence of antibodies to T. gondii in the complex wild boars, hunting dogs and hunters, and carries out an epidemiological risk analysis.

**Changed:** The sentence has been changed.

**Now you read: “**This is the first study that reposts the presence of antibodies to T. gondii in the complex wild boars, hunting dogs and hunters, and carries out an epidemiological risk analysis.” (Page 15, Lines 274-276).

1.15. Line, 411 *T. gondii*

**Changed:** “*Toxoplasma gondii*” has been changed to “*T. gondii*”.

**Now you read: “**The present study is the first report of concomitant exposure to *T. gondii* in wild boars, hunting dogs, and hunters worldwide to date.” (Page 20, Lines 400-401).

**Reviewer #2:**

**Comments to the Author**

1. If the authors have adequately addressed your comments raised in a previous round of review and you feel that this manuscript is now acceptable for publication, you may indicate that here to bypass the “Comments to the Author” section, enter your conflict of interest statement in the “Confidential to Editor” section, and submit your "Accept" recommendation.

Reviewer #2: All comments have been addressed

2. Is the manuscript technically sound, and do the data support the conclusions?

Reviewer #2: (No Response)

3. Has the statistical analysis been performed appropriately and rigorously?

Reviewer #2: Yes

4. Have the authors made all data underlying the findings in their manuscript fully available?

Reviewer #2: Yes

5. Is the manuscript presented in an intelligible fashion and written in standard English?

Reviewer #2: Yes

**Review Comments to the Author**

The article has been significantly improved. I accept all authors answers and explanations.

In this form is ready for publication.

**Answer:** We appreciate the positive comment on the study.
