# Supplementary material for: Seroprevalence of anti-Toxoplasma gondii antibodies in wild boars (Sus scrofa), hunting dogs, and hunters of Brazil
Source: PLoS One. 2019 Oct 11;14(10):e0223474. doi: 10.1371/journal.pone.0223474 (PMC6788692; doi:10.1371/journal.pone.0223474)
Supplement: Attachment — Submitted filename: Response to reviewers 19thSept2019.docx [file pone.0223474.s005.docx]

Curitiba, Brazil, September 19^th^, 2019

**Response to reviewers**

To: Paulo Lee Ho, PhD / Academic Editor - PLOS ONE

Re: author’s PLOS ONE manuscript resubmission (PONE-D-19-16414)

Dear Dr. Ho,

Please find attached our reviewed manuscript entitled " Seroprevalence of anti-*Toxoplasma gondii* antibodies in wild boars (*Sus scrofa*), hunting dogs, and hunters of Brazil " (PONE-D-19-16414). All the corrections and suggestions indicated by the reviewers were fully accepted, addressed and rewritten.

A letter has been written to pinpoint the reviewers’ requests, with a detailed list of corrections and a description of changes made within the manuscript. To better identify the corrections of each reviewer, changes were highlighted in different colors in the revised manuscript.

Thank you for the opportunity and please don’t hesitate to contact us with any further question.

Sincerely,

Alexander Welker Biondo, DMV, MSc, PhD.

Professor, Department of Veterinary Medicine, UFPR, Brazil

Visiting Professor, Purdue University, IN, USA

E-mail: [abiondo@ufpr.br](mailto:abiondo@ufpr.br) / Phone: +55 (41) 3350-5812

**Manuscript PONE-D-19-16414**

**Title:** "Seroprevalence of anti-*Toxoplasma gondii* antibodies in wild boars (*Sus scrofa*), hunting dogs, and hunters of Brazil."

**Dear Reviewer #1,**

Firstly, we appreciate all your suggestions and comments and our manuscript. Certainly, your suggestions were very important to improve the manuscript.

**1. Reviewer #1 (green intext):**

Comments to the Author

1. If the authors have adequately addressed your comments raised in a previous round of review and you feel that this manuscript is now acceptable for publication, you may indicate that here to bypass the “Comments to the Author” section, enter your conflict of interest statement in the “Confidential to Editor” section, and submit your "Accept" recommendation.

Reviewer #1: (No Response)

2. Is the manuscript technically sound, and do the data support the conclusions?

The manuscript must describe a technically sound piece of scientific research with data that supports the conclusions. Experiments must have been conducted rigorously, with appropriate controls, replication, and sample sizes. The conclusions must be drawn appropriately based on the data presented.

Reviewer #1: Yes

3. Has the statistical analysis been performed appropriately and rigorously?

Reviewer #1: Yes

4. Have the authors made all data underlying the findings in their manuscript fully available?

Reviewer #1: Yes

5. Is the manuscript presented in an intelligible fashion and written in standard English?

Reviewer #1: Yes

7. PLOS authors have the option to publish the peer review history of their article (what does this mean?). If published, this will include your full peer review and any attached files.

If you choose “no”, your identity will remain anonymous but your review may still be made public.

Do you want your identity to be public for this peer review? For information about this choice, including consent withdrawal, please see our Privacy Policy.

Reviewer #1: No

**Review Comments to the Author (green intext):**

1.1. Line 274: *T. gondii*

**Change:** *T. gondii* has been italicized

**Now you read: “**This is the first study that reposts the presence of antibodies to *T. gondii* in the complex wild boar, hunting dog and hunter, and carries out an epidemiological risk analysis.” (Page 15, Lines 274-276).

1.2.Line 275: the complex wild boar, hunting dog and hunter

**Change:** The sentence has been changed.

**Now you read:** **“**This is the first study that reposts the presence of antibodies to *T. gondii* in the complex wild boar, hunting dog and hunter, and carries out an epidemiological risk analysis.” (Page 15, Lines 274-276).

1.3.Line 313: *T. gondii*

**Changed: “***Toxoplasma*” has been replaced by “*T. gondii*”

**Now you read:** ”On the other hand, no in-park pets have been allowed in the state park area, and only a dozen ocelots (*Felis pardalis*) and a couple of mountain lions (*Puma concolor*) were observed within the state park limits; these felid species have not yet been confirmed as *T. gondii* definitive hosts or capable of shedding oocysts [1].” (Page 16, Lines 310-314).

1.4.Line 339: *T. gondii*

**Changed: “***Toxoplasma*” has been replaced by “*T. gondii*”

**Now you read: “**In the present study, consumption of raw rat meat had significantly increased the risk of dog seropositivity, which is in agreement with a previous seroprevalence study, where dog contact with rats was associated with a higher risk of *T. gondii* infection [19].” (Page 17, Lines 336-339).
